# Case Report: Macrophage activation syndrome due to multifocal tuberculosis in an immunocompromised patient

**DOI:** 10.12688/f1000research.158982.2

**Published:** 2025-04-17

**Authors:** Salma Riahi, Sana Ammar, Houssem Hassen, Emna Souilem, Donia Mbarki, Yosra Dhaha, Mehdi Ksiaa, Amina Bouatay

**Affiliations:** 1 University of Sousse Faculty of Medicine of Sousse, Sousse, Sousse, Tunisia; 2 Laboratory of Hematology, Sahloul Hospital, Sousse, Sousse, Tunisia; 3 University of Monastir Faculty of Pharmacy of Monastir, Monastir, Monastir, Tunisia; 4 University of Monastir Faculty of Medicine of Monastir, Monastir, Monastir, Tunisia; 5 Department of Gastroenterology, Sahloul Hospital, Sousse, Sousse, Tunisia

**Keywords:** Macrophage activation syndrome, Multifocal tuberculosis, Immunosuppressive treatement.

## Abstract

Macrophage Activation Syndrome (MAS) is a serious and life-threatening complication defined by excessive immune activation. While it’s commonly associated with rheumatic diseases, infections can also trigger MAS, with tuberculosis being a rare but significant cause. This case report discusses a rare occurrence of Macrophage Activation Syndrome (MAS) caused by multifocal tuberculosis in an immunocompromised patient with Crohn’s disease receiving immunosuppressive treatment. The patient is a 26-year-old woman with Crohn’s disease who is being treated with azathioprine. She arrived at the hospital battling persistent abdominal pain, overwhelming fatigue, and fever. Upon examination, splenomegaly and ascites were noted. A chest X-ray revealed bilateral pleural effusion consistent with tuberculosis. A CT scan confirmed the presence of pleural, pericardial, and intraperitoneal fluid. Blood tests indicated pancytopenia, hyperferritinemia, and hypofibrinogenemia. The analysis of ascitic fluid suggested an exudate. The PCR test of the bone marrow aspirate was positive for tuberculosis without rifampicin resistance, and the smear showed hemophagocytosis images. The patient was diagnosed with Macrophage Activation Syndrome secondary to multifocal tuberculosis. This report delves into the complex relationship between MAS and tuberculosis, emphasizing the challenges in diagnosing MAS in such cases and the potential link to tuberculosis. The complex diagnostic landscape of multifocal tuberculosis, which can often mimic malignancies, underscores the importance of promptly detecting and starting anti-tuberculosis interventions for improved clinical outcomes and the prevention of associated complications.

## Introduction

Macrophage Activation Syndrome (MAS) is a rare but life-threatening disease characterized by excessive activation and proliferation of macrophages and T lymphocytes, leading to a cytokine storm and multi-organ dysfunction. ^1^ While MAS is most commonly associated with rheumatic diseases, mainly systemic juvenile idiopathic arthritis and adult-onset Still’s disease, it can also be triggered by infections, malignancies, and certain medications. ^2^ However, it is rarely reported as a complication of tuberculosis, especially in immunocompromised patients. The clinical presentation of MAS is generally brutal, associating persistent fever with bi or pancytopenia. The diagnosis of MAS can be challenging, as its clinical and laboratory features may overlap with those of the underlying disease or infection. ^3^ We present a case of a 26-year-old woman treated with azathioprine for her Crohn’s disease complicated with multifocal tuberculosis and MAS.

## Case presentation

**Figure 1.**
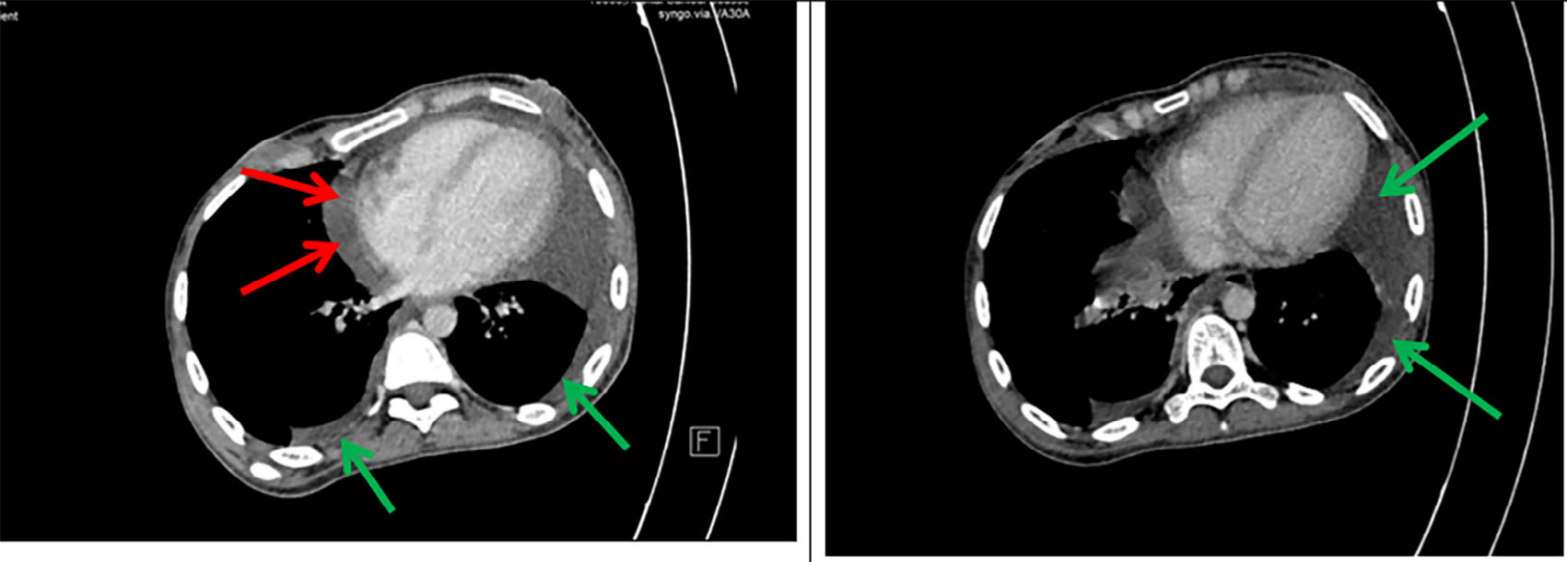
Axial contrast-enhanced CT scan of the chest shows bilateral low abundance pleural effusion (green arrows) and low abundance pericardial effusion (red arrows).

A 26-year-old- woman was referred to our hospital with abdominal pain for five days, fatigue, and fever (39°C). Her medical history included Crohn’s disease diagnosed at the age of 21 with inflammation located in the terminal ileum, initially treated with corticosteroids followed by azathioprine as maintenance therapy. The patient declared no exposure history to active tuberculosis in the past. Her heart rate was 121 beats per minute, blood pressure 110/77 mmHg, respiratory rate 25 breaths per minute, and temperature 39°C. The patient had splenomegaly without hepatomegaly or adenopathy. Shifting dullness and a positive fluid wave test were found. No other signs were reported on physical examination. A chest X-ray was performed, showing bilateral pleural effusion compatible with tuberculosis. An abdominal CT scan was conducted, revealing the presence of both pleural effusion and a significant volume of ascites. High axial sections through the thoracic base showed bilateral pleural and pericardial effusion ( Figure 1).

**Figure 2.**
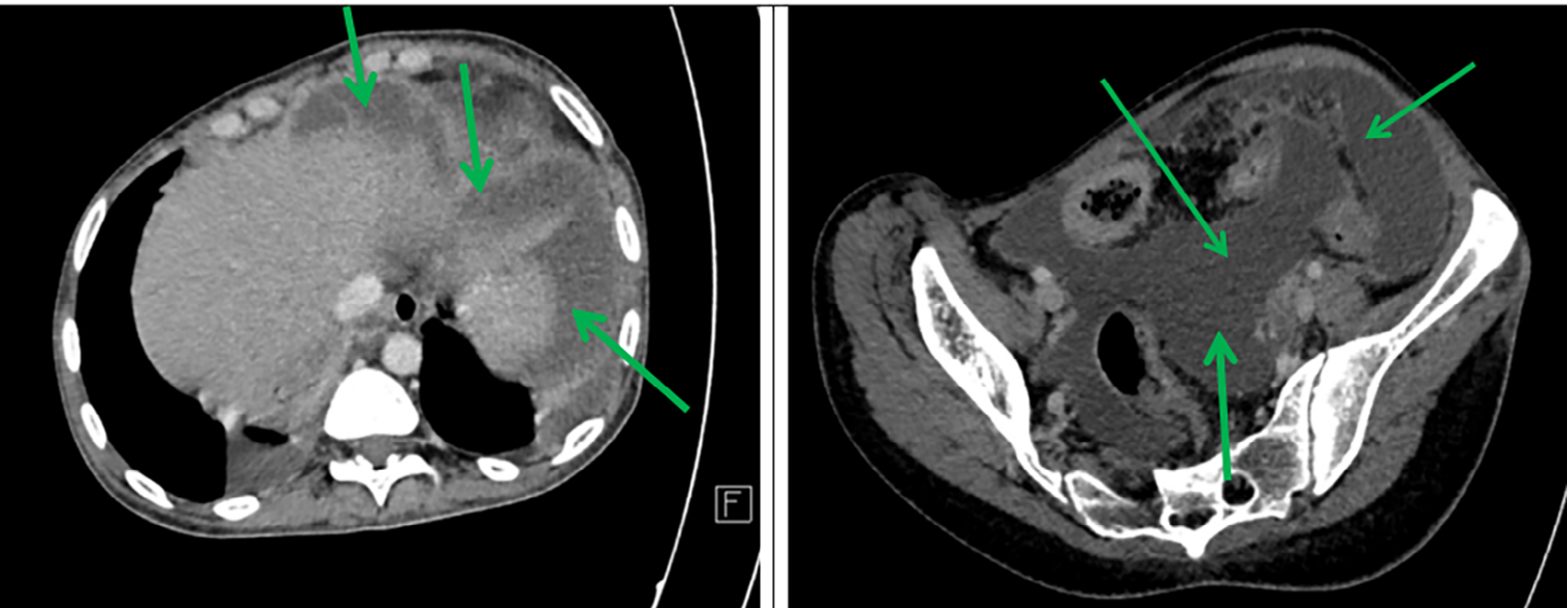
Axial contrast-enhanced abdominal CT scan after contrast injection showing abundant peritoneal effusion (green arrows).

Axial sections taken at portal time after injection of contrast agent showed abundant intra-peritoneal effusion ( Figure 2).

A complete blood count showed normochromic normocytic anaemia, neutropenia, lymphopenia, and thrombocytopenia ( Table 1).

**Table 1.** The Hemogram results.

Hematology parameters	Patient values on admission	Patient values on Day 1	Patient values on Day 7	Patient values after treatment completion	Reference ranges
Hemoglobin (g/dL)	7.6	6.9	8.7	13.9	Male: 13–18 Female: 12.0–16.0
WBC (cell/mm ^3^)	1400	1300	2900	6200	4500–11,000
Neutrophils (cell/mm ^3^)	1200	1100	2400	4000	1500–8000
Lymphocytes (cell/mm ^3^)	100	100	300	1400	1000–4000
Platelets (cell/mm ^3^)	109,000	98,000	198,000	216,000	150,000–400,000

WBC: White Blood Cells.

The patient had elevated lactate dehydrogenase (LDH: 670 UI/L), D-dimer (7.017 μg/ml), prolonged prothrombin time (PT: 61%), and activated partial thromboplastin time (aPTT: 37.7 sec, ratio M/T = 1.3). Additionally, there was hyperferritinemia (1050 ng/mL), and low Fibrinogen level (1.5 g/L). Mild liver cholestasis was noticed (gamma-glutamyl transpeptidase (GGT) at 52 UI/L; alkaline phosphatase (ALP) at 184 UI/L). The triglyceride level was normal (0.8 mmol/L) and the electrolyte test showed hyponatremia (125 mmol/L). The low serum Ascites Albumin Gradient (SAAG) (<1.1g/dL) suggested that the ascitic fluid is an exudate. Fluid’s direct microscopic examination revealed no tuberculosis bacilli and the acid-fast stain was negative. Unfortunately, cultures and PCR studies were not performed on pleural and ascitic fluids due to resource limitations, as PCR testing is not always available in our facility. No renal function abnormality was found. Viral serology HIV, HSV, HBV, HCV, EBV, and CMV were negative. Azathioprine toxicity was not ruled out, as testing was not available in our facility. An immune workup was performed, including antinuclear antibody (ANA) testing, which was negative, and an immunoglobulin assay, which was normal. There were no clinical signs suggestive of congenital immunodeficiency, but no further immunological tests were carried out due to resource limitations.

**Figure 3.**
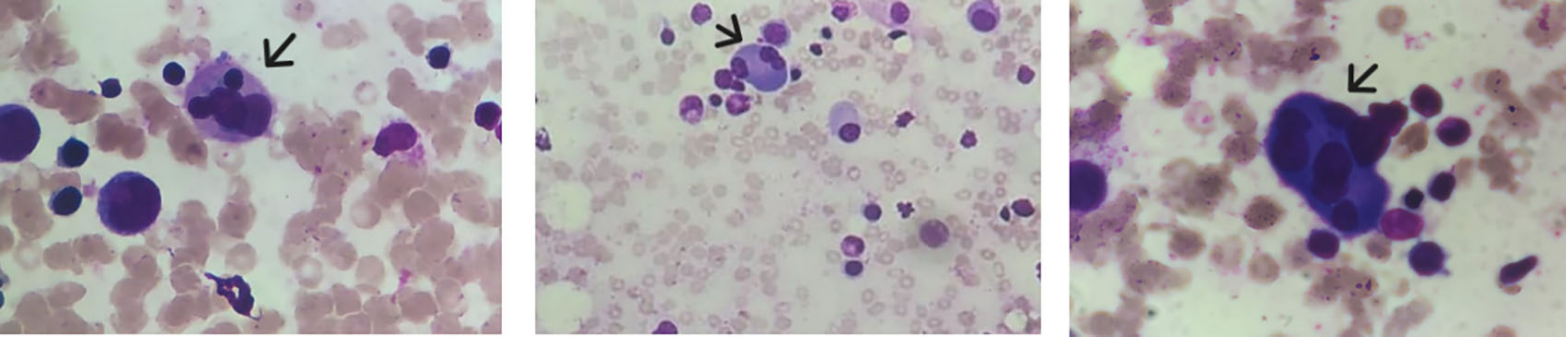
Bone marrow aspirate smear colored with MGG staining displays features of haemophagocytosis with erythroids engulfed by macrophages (Black arrows).

The febrile cytopenia associated with the splenomegaly led to a bone marrow aspirate; an Xpert MTB/RIF PCR was performed and turned out positive. Rifampicin resistance was not detected. The bone marrow aspirate smear showed a reactional plasmocytosis and multiple hemophagocytosis images ( Figure 3).

Bone Marrow Biopsy was not performed. The diagnosis of macrophage activation syndrome (MAS) was established based on clinical and laboratory criteria. Given the clinical presentation and laboratory findings, multifocal tuberculosis complicated by MAS was diagnosed in an immunocompromised patient. The patient was started on a standard daily oral anti-tuberculosis regimen, including isoniazid, rifampicin, pyrazinamide, and ethambutol hydrochloride.

The patient did not receive corticosteroids or other immunomodulatory therapy beyond anti-tuberculosis treatment. Apyrexia was achieved within 72 hours of treatment initiation, and there was a progressive improvement in platelet count after one week (Table 1). The fibrinogen level increased to 2.02 g/L by day 7, while LDH decreased to 352 UI/L, PT improved to 70%, and aPTT to 35.4 sec (ratio M/T = 1.22). No adverse effects of anti-tuberculosis treatment were observed.

The patient was discharged to continue treatment at home. At follow-up, there was radiological resolution of pleural and ascitic effusions at the end of therapy, and a normal blood count was observed ( Table 1).

## Discussion

Tuberculosis (TB) remains a significant cause of death globally. According to WHO, there were approximately 10.6 million new TB cases in 2021. ^4^ TB is endemic in Tunisia, with an estimated incidence of 22.46/100,000 inhabitants in 2021. ^5^

It is widely recognized that immunosuppressive therapy elevates the risk of tuberculosis, particularly in endemic regions like Tunisia. ^6^ A study by Fortes et al. assessed 301 patients with inflammatory bowel disease (IBD) and found that azathioprine treatment increased the risk of developing active tuberculosis by 6.87 times compared to patients who were not receiving this medication. ^7^ In our case, the patient had Crohn's disease diagnosed at the age of 21, initially treated with corticosteroids, followed by azathioprine as maintenance therapy. MAS is a serious medical condition that can be triggered by infectious agents, particularly certain viruses and mycobacteria. The majority of cases involving tuberculosis complicated by MAS occur in individuals who are immunocompromised such as transplant recipients, cancer patients, or those receiving immunosuppressive therapy. ^8^ The disease presents significant challenges and is often associated with a poor prognosis. However, it is essential to approach this matter with a focus on early diagnosis and targeted treatment options, which can lead to improved outcomes and better management of the condition. In our case, MAS was due to multifocal tuberculosis, which involves multiple systems with associated symptoms, making the diagnosis challenging.

Multifocal tuberculosis is characterised by large multifocal tuberculosis areas in the same or different adjacent or distant organs; in our case, the organs concerned were the lungs, the digestive system, and the hematopoietic organs. ^9^ Regarding microbiological investigations, cultures and PCR studies were not performed on pleural and ascitic fluids due to resource constraints. However, a bone marrow aspirate was analyzed, and PCR for Mycobacterium tuberculosis (Xpert MTB/RIF) was positive, confirming the diagnosis. This highlights the challenges faced in settings with limited access to advanced diagnostic tools, where a rapid clinical assessment remains crucial for timely diagnosis and management. Especially when the prognosis is severe, with a mortality rate of 16 to 25%, depending on the author. ^10, 11^

Sporadic cases of multifocal tuberculosis have been reported; most of them presented with anaemia, leucopenia, and/or thrombocytopenia. In our case, the patient had pancytopenia, which could have been multifactorial. While azathioprine toxicity was considered, we were unable to definitively rule it out due to the unavailability of specific diagnostic tests in our facility.

MAS is an acute, sometimes fatal complication of multifocal tuberculosis. The clinical presentation is usually non-specific and can be misleading. MAS is a condition associated with hemophagocytic lymphohistiocytosis (HLH), which is categorized into primary and secondary HLH. Primary HLH is a genetic disorder, while secondary HLH arises from other conditions, such as cancers, autoimmune disorders, and infections like tuberculosis, as seen in this instance. EBV is the most prevalent infectious cause, but MAS can also be related to various infections, including viral (HIV, CMV), bacterial (Salmonella typhi), and parasitic (Leishmania sp., Toxoplasma gondii) origins. ^12^

There is a set of clinical and biological criteria to define a MAS, but according to the literature, some remain more important than others. The most used criteria were Hepatosplenomegaly, fever, cytopenia, hypofibrinogenemia, hyperferritinemia, hypertriglyceridemia, hemophagocytosis, and hepatopathy. Six out of all the criteria cited below ( Table 2) were found in our patient. ^13^

**Table 2.** Diagnostic criteria of hemophagocytic lymphohistiocytosis: HLH-2004. ^14^

Diagnosis will be established if one of either (1) or (2) is fulfilled
(1) Molecular diagnosis consistent with HLH (2) Diagnostic criteria for HLH fulfilled (5 out of the 8 criteria shown below) - Fever ≥38.5°C for ≥7 days - Splenomegaly ≥3 finger breadth below the left subcostal margin - Cytopenias affecting ≥2 of 3 lineages in peripheral blood • Hemoglobin <9 g/L • Platelets <100×109/L • Absolute neutrophil count <1.0×109/L - Hypertriglyceridemia and/or hypofibrinogenemia Fasting triglycerides ≥265 mg/dL, Fibrinogen ≤1.5 g/L - Hemophagocytosis in the bone marrow or spleen or lymph node - Low or absent NK cell activity (according to the local laboratory reference) - Ferritin ≥500 μg/L - Soluble CD25 (sIL-2 receptor) ≥2,400 U/mL

Abbreviations: HLH, hemophagocytic lymphohistiocytosis; NK, natural killer; sIL-2, soluble interleukin-2.

A study examined 37 documented instances of tuberculosis linked to MAS, revealing that half of the patients were immunocompromised (due to renal transplants, cancer, or HIV). The clinical symptoms remained consistent (such as fever and hepatosplenomegaly). In 80% of the cases, it was a disseminated tuberculosis. ^15^

In most bone marrow aspirations, cellularity appears quite normal, and hemophagocytic histiocytes typically account for 2 to 75% of total nucleated cells. The mortality rate associated with MAS and tuberculosis reaches 50%, rising to 100% for those who do not receive any antituberculous treatment. ^16^

The observed therapeutic response in our patient was characterized by the resolution of fever within 72 hours and improvement in platelet count after one week of initiating anti-tuberculosis therapy without additional immunomodulatory treatment suggesting a direct impact of Mycobacterium tuberculosis on bone marrow suppression rather than an autoimmune process. Kalra et al. reported a case of immune thrombocytopenia associated with disseminated tuberculosis, in which the patient required both anti-tuberculosis therapy and corticosteroids to achieve platelet normalization. ^17^ This suggests a distinction from immune-mediated thrombocytopenia, where corticosteroids are often required for platelet recovery.

## Conclusion

In conclusion, we would like to present a rare case of MAS triggered by multifocal tuberculosis in a Crohn’s disease patient receiving immunosuppressive therapy. MAS is a severe complication that necessitates prompt diagnosis and management. Our case underscores the significance of considering atypical infectious symptoms in immunocompromised patients and promptly suspecting tuberculosis, particularly in patients exhibiting signs of MAS. Timely diagnosis and initiating antitubercular therapy are imperative for enhancing outcomes and averting complications.

## Ethical consideration

Ethical approval was not required.

## Consent statement to Publish

Written informed consent was obtained from the patient upon admission, permitting the use of their de-identified data for research and publication purposes. All identifying information has been removed to ensure the patient’s confidentiality following ethical guidelines.

## Data Availability

No data associated with this article.
