# Supplementary material for: Case Report: Macrophage activation syndrome due to multifocal tuberculosis in an immunocompromised patient
Source: F1000Res. 2025 Apr 17;13:1439. Originally published 2024 Nov 27. [Version 2] doi: 10.12688/f1000research.158982.2 (PMC12012428; doi:10.12688/f1000research.158982.2)
Supplement: Peer review history

# Reviewer report: Reviewer response for version 2

**DOI:** 10.5256/f1000research.180247.r378709

**Published:** 2025-04-21

**Authors:** Luisa Berenise Gámez-González

**Affiliations:** 1 Autonomous University of Chihuahua, Chihuahua, Mexico

The manuscript has been adequately improved, and all of my suggestions and concerns have been addressed. I have no further modifications to suggest.

Are enough details provided of any physical examination and diagnostic tests, treatment given and outcomes?

Partly

Is the case presented with sufficient detail to be useful for other practitioners?

Partly

Is sufficient discussion included of the importance of the findings and their relevance to future understanding of disease processes, diagnosis or treatment?

Partly

Is the background of the case’s history and progression described in sufficient detail?

Partly

Reviewer Expertise:

Clinical Immunology

I confirm that I have read this submission and believe that I have an appropriate level of expertise to confirm that it is of an acceptable scientific standard.

# Reviewer report: Reviewer response for version 2

**DOI:** 10.5256/f1000research.180247.r378710

**Published:** 2025-04-18

**Authors:** W Winn Chatham

**Affiliations:** 1 University of Nevada Las Vegas, Las Vegas, Nevada, USA

This is now fine to publish as is - authors have sufficiently addressed the initial criticisms.

Are enough details provided of any physical examination and diagnostic tests, treatment given and outcomes?

No

Is the case presented with sufficient detail to be useful for other practitioners?

No

Is sufficient discussion included of the importance of the findings and their relevance to future understanding of disease processes, diagnosis or treatment?

No

Is the background of the case’s history and progression described in sufficient detail?

Yes

Reviewer Expertise:

Systemic Lupus, Macrophage activation syndromes, Immunodeficiency associated autoimmunity

I confirm that I have read this submission and believe that I have an appropriate level of expertise to confirm that it is of an acceptable scientific standard.

# Reviewer report: Reviewer response for version 1

**DOI:** 10.5256/f1000research.174644.r357485

**Published:** 2025-02-03

**Authors:** Luisa Berenise Gámez-González

**Affiliations:** 1 Autonomous University of Chihuahua, Chihuahua, Mexico

It would be relevant to mention whether an immune workup was performed to rule out an underlying autoimmune or primary immunodeficiency condition, given the patient’s immunosuppressed state and the development of MAS.

 It would be valuable to specify whether the patient required additional immuno-modulatory treatment (e.g., corticosteroids, ) beyond anti-tuberculosis therapy. Additionally, a more detailed description of the clinical course, including the time to improvement and resolution of MAS-related complications, would strengthen the discussion

 I suggest specifying the age at which Crohn’s disease was diagnosed instead of the year, as this provides clearer clinical context.

Are enough details provided of any physical examination and diagnostic tests, treatment given and outcomes?

Partly

Is the case presented with sufficient detail to be useful for other practitioners?

Partly

Is sufficient discussion included of the importance of the findings and their relevance to future understanding of disease processes, diagnosis or treatment?

Partly

Is the background of the case’s history and progression described in sufficient detail?

Partly

Reviewer Expertise:

Clinical Immunology

I confirm that I have read this submission and believe that I have an appropriate level of expertise to confirm that it is of an acceptable scientific standard, however I have significant reservations, as outlined above.

# Response

**Published:** 2025-04-03

**Authors:** Salma Riahi

We sincerely appreciate your insightful comments and suggestions, which have helped us refine our manuscript. Below, we address each point raised:

1. **Immune Workup for Autoimmune or Primary Immunodeficiency Conditions**
  We acknowledge the importance of assessing underlying immune disorders in the context of MAS. In this case, the immune workup was limited to antinuclear antibody (ANA) testing, which was negative, and an immunoglobulin test, which was normal. Due to resource limitations, further immunological investigations were not performed. However, there were no clinical or biological signs strongly suggestive of a primary immunodeficiency or additional autoimmune condition beyond Crohn’s disease and its treatment-related immunosuppression. We will clarify this point in the revised manuscript.
2. **Additional Immunomodulatory Treatment**
  The patient did not receive corticosteroids or other immunomodulatory therapy beyond anti-tuberculosis treatment. The clinical decision was based on the patient’s overall condition and response to tuberculosis therapy. We will specify this in the revised discussion.
3. **Clinical Course and Time to Improvement**
  A more detailed description of the patient’s evolution will enhance the discussion. In the revised manuscript, we will provide a clearer timeline of the resolution of MAS-related complications and the patient’s response to treatment.
4. **Age at Crohn’s Disease Diagnosis**
  We appreciate this suggestion and will specify the patient’s age at diagnosis instead of the year to provide a clearer clinical context.

Thank you again for your valuable feedback. We believe these revisions will strengthen our manuscript.

# Reviewer report: Reviewer response for version 1

**DOI:** 10.5256/f1000research.174644.r347815

**Published:** 2024-12-30

**Authors:** W Winn Chatham

**Affiliations:** 1 University of Nevada Las Vegas, Las Vegas, Nevada, USA

This is a brief case report describing a patient diagnosed with disseminated tuberculosis who had features of associated macrophage activation syndrome, a previously recognized association.

 Comments:

 As written, this report adds little if anything to existing knowledge. There are no apparent unique features of the case or relevant emphasized instructive teaching points. 

 Perhaps discussions of the outcome of this case (no information was provided) would provide some instructive insights. What exactly is meant by "MAS was retained"? Did the MAS features abate with anti-tuberculous therapy and if so, what was the time frame?

 Missing relevant data:

 Were cultures or PCR studies performed and positive on the pleural and ascites fluids?

 As the fibrinogen was only borderline abnormal, were there other features of coagulopathy (elevated serum LDH, d-dimer, elevated PT/INR)?

 Was azathioprine toxicity considered or ruled out as a cause of the pancytopenia?

Are enough details provided of any physical examination and diagnostic tests, treatment given and outcomes?

No

Is the case presented with sufficient detail to be useful for other practitioners?

No

Is sufficient discussion included of the importance of the findings and their relevance to future understanding of disease processes, diagnosis or treatment?

No

Is the background of the case’s history and progression described in sufficient detail?

Yes

Reviewer Expertise:

Systemic Lupus, Macrophage activation syndromes, Immunodeficiency associated autoimmunity

I confirm that I have read this submission and believe that I have an appropriate level of expertise to state that I do not consider it to be of an acceptable scientific standard, for reasons outlined above.

# Response

**Published:** 2025-04-03

**Authors:** Salma Riahi

We sincerely appreciate your thoughtful review of our manuscript and your valuable suggestions. Below, we address the points raised and outline the revisions made to improve the clarity and relevance of our case report.

1. **Outcome and Evolution Under Anti-Tuberculosis Therapy**
  We acknowledge the importance of providing details on the patient’s clinical course. Our revised manuscript will include a detailed discussion on the evolution of the patient’s condition, highlighting how the MAS features evolved under anti-tuberculosis treatment and the time frame of clinical and biological improvements.
2. **Clarification of "MAS was retained"**
  We recognize that this phrasing may be unclear. In the revised version, we will clarify that the diagnosis of MAS was established based on clinical and laboratory criteria and elaborate on how the condition evolved after initiating anti-tuberculosis treatment.
3. **Additional Diagnostic Data**
  - **Cultures and PCR Studies:** Unfortunately, due to resource limitations, cultures and PCR studies were not performed on pleural and ascitic fluids. PCR testing is not always available in our facility. We will clarify this limitation in the revised manuscript.
  - **Coagulopathy Markers:** While fibrinogen was only borderline abnormal, we will include additional coagulation markers (serum LDH, D-dimer, PT/INR) if available in our records to further assess coagulopathy.
  - **Azathioprine Toxicity:** We acknowledge that azathioprine toxicity is a differential diagnosis for pancytopenia. However, due to limited laboratory resources in our facility, we were unable to promptly confirm or rule out this possibility. We will explicitly mention this limitation in the revised discussion.
4. **Significance of the Case Report**
  We appreciate the concern regarding the novelty of our case. This report's main contribution is to emphasize the importance of early recognition and diagnosis of MAS in disseminated tuberculosis, particularly in resource-limited settings where certain diagnostic tests may not be readily available. We will strengthen this point in our discussion to highlight the clinical relevance of our case.

Thank you again for your constructive feedback. We believe these revisions will enhance the clarity and impact of our manuscript.
